# Can Red Blood Cell and Platelet Parameters Be Associated With Inflammation in Children With Tic Disorder?

**DOI:** 10.7759/cureus.47280

**Published:** 2023-10-18

**Authors:** Mahmut Zabit Kara, Müslüm Kul

**Affiliations:** 1 Child and Adolescent Psychiatry, University of Health Sciences, Antalya Training and Research Hospital, Antalya, TUR; 2 Child and Adolescent Psychiatry, Mersin City Training and Research Hospital, Mersin, TUR

**Keywords:** hematocrit, psychiatric disorder, tourette's syndrome, provisional tic disorder, neurodevelopmental disorder, mean corpuscular volume, platelet count (plt), complete blood count (cbc), tic disorder

## Abstract

Objective: Tic disorder (TD) is one of the neurodevelopmental disorders and its etiology has not been fully elucidated. Complete blood count (CBC) values have been used as indicators of a systemic inflammatory response. In our study, we aimed to assess hemogram parameters in drug-naive, comorbidity-free children with TD compared with controls.

Methods: This retrospective study included 62 drug-naive children with TD who had undergone CBC within one month prior to the study. A control group of 48 healthy children, matched for age and gender, without any organic or psychiatric disorders, was included. Statistical analysis was performed by using IBM SPSS Statistics for Windows, Version 22.0 (Released 2013; IBM Corp., Armonk, New York, United States).

Results: Hematocrit (p = 0.044), mean corpuscular volume (p = 0.002), platelet count (p = 0.011), and plateletcrit (p = 0.031) values were significantly higher in the TD group, whereas mean corpuscular hemoglobin concentration (p = 0.00) was significantly lower in the TD group. Additionally, a significant negative correlation was observed between the duration of illness and platelet (p = 0.05, r=-0.282), plateletcrit (p = 0.038, r = -0.295), and neutrophil count (p = 0.006, r = -0.391), while a positive correlation was found between the duration of illness and eosinophil count (p = 0.018, r = 0.336).

Conclusion: The results revealed several significant differences in hemogram parameters between TD patients and the control group. These may suggest the role of inflammation and/or other underlying mechanisms in TD and may inspire new studies. Future studies with larger and more homogeneous samples, including comprehensive inflammatory markers, may contribute to a deeper understanding of the relationship between inflammation and TD.

## Introduction

Tic disorders (TD) are a group of neurodevelopmental disorders with onset in childhood that are marked by sudden, rapid, recurrent, non-rhythmic movements and/or vocalizations. Primary TD mainly includes Tourette's syndrome (TS), provisional tic disorder, and chronic motor or vocal tic disorder [1]. Their clinical presentations are varied, and they are frequently accompanied by psychopathological and/or behavioral comorbidities such as attention-deficit/hyperactivity disorder (ADHD), obsessive-compulsive disorder (OCD), anxiety, depression, and sleep disturbances [2]. Epidemiological studies have shown that TD occurs at a similar rate across all racial and ethnic groups, affecting approximately 20% of school-age children with mild and temporary tics [3]. In various Western populations, chronic TD affects 0.3-5.0% of school-age children, while TS affects about 0.3-1.0% [2].

Today, the etiology and pathogenesis of TDs in children are not fully clarified, but it is generally believed that these disorders result from a combination of genetic, environmental, and psychological factors [4]. Additionally, there are an increasing number of studies on the potential involvement of immunological mechanisms in TDs. The relation between Group A β-hemolytic streptococci infections and TD onset or exacerbation in some children shows that immunological factors play a role in the etiology of TD [5]. Apart from *Streptococcus* infection, other infections by *Mycoplasma pneumoniae*, enterovirus, *Chlamydia pneumoniae*, *Borrelia burgdorferi*, *Toxoplasma gondii*, coronavirus disease 2019 (COVID-19) and even human immunodeficiency virus (HIV) have been related with TD [6,7]. In addition, it has been found that TD is associated with common allergic diseases, including rhinitis, asthma, dermatitis, and allergic conjunctivitis [8]. TD and allergic diseases both show abnormalities in inflammatory indicators such as interleukins and tumour necrosis factor (TNF) [9]. In studies where immunological parameters are directly evaluated, abnormal peripheral immune activities such as decreased regulatory cells, increased release of pro-inflammatory cytokines such as TNF-alpha and IL-12, activation of T cells and B cells, and decreased IgG3 level have been demonstrated [10-12]. Studies on brain tissue and cerebrospinal fluid showed that altered microglial activation and increased oligoclonal bands of IgG may result in neuro-inflammation in TD [10,13]. These findings collectively suggest that immune dysregulation may play a central role in the development of TD.

In the existing literature, researchers have explored various aspects of inflammatory processes in TD. However, the methods employed in these studies are often complex and expensive, making them less practical for routine clinical use. On the contrary, recent publications have examined several complete blood count (CBC) parameters to assess inflammatory states in both organic and psychiatric disorders. These investigations have been carried out on neurodevelopmental conditions like autism spectrum disorder (ASD), ADHD, and stuttering, as well as psychiatric disorders such as OCD [14-17]. The key focus while studying these parameters is their ease of use and cost-effectiveness in everyday clinical practice.

To the best of our knowledge, there hasn't been any research specifically investigating hemogram parameters in individuals with TD. In this study, we aimed to evaluate the hemogram parameters of drug-naive and comorbidity-free TD patients by comparing with controls to determine the role of inflammation in TD.

## Materials and methods

In this retrospective study, we reviewed the medical files of children with TD who were admitted to the Child and Adolescent Psychiatry Department of Antalya Training and Research Hospital, Antalya, Türkiye, between May 2020 and May 2023. The study was approved by the Ethics Committee of Antalya Training and Research Hospital (Protocol Number: 10/3, dated March 28, 2019).

The Kiddie Schedule for Affective Disorders and Schizophrenia-Present and Lifetime Version (K-SADS-PL) is a semi-structured diagnostic tool used to assess various psychiatric disorders, including emotional and psychotic disorders, as well as tic disorders. The majority of items in the K-SADS-PL are scored using a 0-3 point rating scale (0 = no information is available, 1 = the symptom is not present, 2 = sub-threshold presentation, and 3 = threshold presentation of symptoms) [18]. The Turkish validity and reliability of K-SADS-PL (K-SADS-PL-T) were conducted by Gokler et al. [19]. The validity of K-SADS-PL-T for tic disorders was rated as good, with excellent interrater and test-retest reliability. The diagnostic interviews were conducted by an experienced child and adolescent psychiatrist using the K-SADS-PL-T. The children with TD who had CBC done within a month prior to the time of assessment were enrolled in the study. Patients with missing file information were not included in the study. Age- and sex-matched healthy children without any psychiatric disorders, as assessed by clinical interviews, were recruited from the healthy child clinic of the hospital concurrently. The exclusion criteria for both groups were as follows: use of psychotropic drugs, psychiatric comorbidity, presence of acute or chronic medical disorder, acute or chronic inflammatory diseases, concomitant use of any medications, abnormal vital signs (e.g. fever), or laboratory test results (e.g. anemia, leukopenia).

Venous blood samples were obtained from the antecubital vein of both patients and controls between 8 a.m. and 10 a.m. after at least eight hours of starving. The samples were centrifuged within 30 minutes and on the same day. Reference intervals were determined as follows: white blood cell (WBC) 4-10.5 (103 /uL), red blood cells (RBC) 4.2-5.3 (106 /uL), hemoglobin 12.5-.16.1 (g/dL), hematocrit (HCT): 36-47 (%), mean corpuscular volume (MCV) 78-95 (fL), mean corpuscular hemoglobin (MCH) 27.2-33.5 (pg), mean corpuscular hemoglobin concentration (MCHC) 31.8-34.2(g/dL), platelet 150-450 (10^3^/uL), mean platelet volume (MPV) 9.6-11.8, platelet distribution width (PDW) 10.1-16.1 (%), plateletcrit (PCT) 0-1 (%), neutrophils 1.54-7.04 (10^3^/uL), lymphocytes 0.97-3.26 (10^3^/uL), monocytes 0.18-0.78 (10^3^/uL), eosinophils 0.04-0.38 (10^3^/uL), basophils 0.02-0.08 (10^3^/uL).

Statistical analysis was performed by using IBM SPSS Statistics for Windows, Version 22.0 (Released 2013; IBM Corp., Armonk, New York, United States). Descriptive statistics were used to define the demographic and clinical variables of all participants. The data are expressed as mean or median as appropriate. The normality of the data distributions was checked with the Kolmogorov-Smirnov test. Student’s t-test was used for normally distributed variables whereas abnormally distributed variables were compared using the Mann-Whitney U test. The Kruskal-Wallis test was conducted to compare variables that were not normally distributed among groups for multiple comparisons. Pearson correlation analysis was performed in the TD group. A p-value of less than 0.05 was considered significant.

## Results

In this study, we examined the medical records of 201 children who had been diagnosed with TD between May 2020 and May 2023. Excluding 139 patients with accompanying physical or psychiatric conditions, with a history of medication use, without a CBC analysis, or with missing file information, a total of 62 patients (42 male and 20 female) were included in the study. The control group comprised 48 healthy children (32 males and 16 females) matched for age and gender. The mean age in the TD group was 11.94±3.37 years, and in the control group, it was 11.02±2.23 years. The gender distribution (p = 0.533) and mean ages (p = 0.092) were statistically similar in both groups.

According to the analysis of hemogram parameters, in the TD group, HCT (p = 0.044), MCV (p = 0.002), platelet (p = 0.011), and PCT (p = 0.031) were found to be significantly higher compared to the controls, while MCHC (p<0.001) was significantly lower in the TD group. Other parameters were found to be similar in both groups. The demographic data of the participants along with the hemogram parameters are shown in Table 1. The duration of TD was 26.85 months.

**Table 1:** Comparison of sociodemographic variables and blood count parameters between the two groups *P < 0.05 WBC: white blood cell; RBC: red blood cell; HCT: hematocrit; MCV: mean corpuscular volume; MCH: mean corpuscular hemoglobin; MCHC: mean corpuscular hemoglobin concentration; MPV: mean platelet volume; RDW_SD: red cell distribution width_standard deviation; PDW: platelet distribution width; PCT: plateletcrit; P-LCR: platelet larger cell ratio, NLR: neutrophil to lymphocyte ratio; MLR: monocyte to lymphocyte ratio; PLR: platelet to lymphocyte ratio

Variable	Reference Range	Tic Disorder Group (n=62), mean±SD	Control Group (n=48), mean±SD	p-value
Age (years)		11.94±3.37	11.02±2.23	0.092
Gender (female/male)		20/42	16/32	0.533
WBC	4-10.5 (10^3^/uL)	8.14±1.79	7.49±1.86	0.070
RBC	4.2-5.3 (10^6^/uL)	4.93±0.39	4.86±0.34	0.342
Hemoglobin	12.5-16.1 (g/dL)	13.18±1.03	13.15±0.94	0.871
HCT	36-47 (%)	39.34±3.19	38.19±2.46	0.044*
MCV	78–95 (fL)	80.07±5.61	78.60±2.79	0.002*
MCH	27.2–33.5 (pg)	26.88±2.24	27.08±1.21	0.576
MCHC	31.8-34.2(g/dL)	33.55±1.23	34.44±1.28	<0.001*
MPV	9.6-11.8	9.91±0.92	10.11±0.85	0.247
RDW_SD	35.1-41.7	37.52±2.66	36.56±2.59	0.069
Platelet	150–450 (10^3^ /uL)	327.10±59.81	294.43±70.12	0.011*
PDW	10.1-16.1 (%)	11.69±2.00	11.13±2.17	0.167
PCT	0–1 (%)	0.32±0.06	0.29±0.05	0.031*
Neutrophil	1.54-7.04 (10^3^ /uL)	4.00±1.40	3.64±1.48	0.203
Lymphocyte	0.97-3.26 (10^3^ /uL)	3.21±1.04	2.94±0.69	0.126
Monocyte	0.18-0.78 (10^3^/uL)	0.61±0.17	0.62±0.15	0.870
Eosinophil	0.04-0.38 (10^3^/uL)	0.25±0.18	0.23±0.17	0.662
Basophil	0.02-0.08 (10^3^/uL)	0.06±0.12	0.04±0.02	0.314
P-LCR		25.18±6.23	25.60±6.56	0.730
NLR		1.47±1.30	1.28±0.54	0.367
MLR		0.23±0.25	0.22±0.07	0.759
PLR		113.83±63.52	103.81±30.06	0.318

In the TD group, the average duration of illness at the time of application was 26.85 months (0.25-120). A significant negative correlation was observed between the duration of illness and platelet (p = 0.021, r = -0.282), PCT (p = 0.038, r = -0.295), and neutrophil (p = 0.006, r = -0.391) while a positive correlation was found between the duration of illness and eosinophil count (p = 0.018, r = 0.336). No significant relationship has been detected between the disease duration and other hemogram parameters (Table 2).

**Table 2:** Pearson’s correlation between duration of tic disorder and complete blood count parameters *Correlation is significant at the 0.05 level (2-tailed); **Correlation is significant at the 0.01 level (2-tailed)

Parameters	Duration of Disorder	
Platelet	r	-0.282^*^
	p	0.021
Plateletcrit	r	-0.295^*^
	p	0.038
Neutrophil	r	-0.391^**^
	p	0.006
Eosinophil	r	0.336*
	p	0.018

## Discussion

In our study, we aimed to evaluate hemogram parameters to understand the inflammation status of children and adolescents with TD by comparing them to the control group. To the best of our knowledge, this is the first study conducted in this regard. The results of our study showed that in TD patients, HCT, MCV, platelet, and PCT values were significantly higher compared to the previous research, certain hematological ol group, while MCHC values were lower in the patient group. Other hemogram parameters were found to be similar in both groups. Negative correlations were observed between the duration of the disease and platelet, PCT, and neutrophil, while a positive correlation was found between the duration of the disease and eosinophil.

Among the parameters related to HCT, the HCT value, which represents the volume of red blood cells, was found to be significantly higher in the TD group. HCT represents the volume of erythrocytes in one unit of whole blood. In a study investigating the relationship between inflammation and HCT, it was observed that HCT was lower in systemic lupus erythematosus patients [20]; it was stated that some patients had anemia due to chronic inflammation. In contrast, in our study, patients with anemia were excluded from the study. The different results obtained in the two studies could be due to differences in the study group and study design. Indeed, in patients with herpes zoster infection, HCT values were higher in the patient group along with other inflammatory parameters [21]. In our study, although RBC and hemoglobin levels were similar between the two groups, a high HCT value may be associated with a possible inflammatory response.

HCT has been studied in a limited number of studies in psychiatric disorders. The high HCT value detected in schizophrenia patients was associated with dehydration and hemodynamic instability due to psychosis [22]. Dehydration and hemodynamic instability observed in psychotic patients have been associated with reduced food and fluid intake due to diminished self-care or delusions of poisoning, excessive sweating due to sympathetic activation caused by psychotic excitement, incessant wandering, or stupor. However, the intravenous and/or intramuscular use of antipsychotics and/or benzodiazepines commonly employed in emergency situations can also lead to serious dehydration [22]. Increased HCT values in depression patients were associated with psychological stress, and it was suggested that this could be related to increased sympathetic system activity and catecholamine release. In the same study, a decrease in HCT was observed after antidepressant treatment [23]. The authors, based on their data obtained through secondary analyses, have proposed the idea that successful antidepressant treatment reduces the psychological symptoms of depression and improves hemorheologic measures of stress-hemoconcentration [23]. Considering the high level of psychological stress in TD patients [4], a mechanism similar to that in depression may be possible in TD as well. Therefore, the elevation of HCT may be related to a possible inflammatory process or a sympathetic system response related to psychological distress. This should be further investigated in future research.

The MCV value was found to be significantly higher in the patient group, in addition to HCT. MCV represents the volume of red blood cells [24]. Elevated MCV levels are associated not only with macrocytic anemias but also with endothelial dysfunction and inflammation [25]. Erythrocytes act as an extracellular antioxidant system due to their capacity to clear exogenous reactive oxygen species (ROS), the permeability of their membranes to oxygen radicals, and high intracellular antioxidant enzyme activities [26]. It is thought that the antioxidant mechanism of erythrocytes may be impaired in the case of increased MCV [25]. Although macrocytosis was not observed in the patient group, increased MCV can be considered indicative of inflammation and immune dysregulation. Consistent with our findings, studies conducted with schizophrenia and depression patients have also reported higher MCV values compared to healthy controls [27,28]. Further research is needed to investigate the relationship between MCV and different inflammatory markers and oxidative stress parameters in TD and other psychiatric disorders.

MCHC is a measure of the concentration of hemoglobin per volume of packed red blood cells. In the TD group, MCHC levels were significantly lower compared to the controls. In a study that investigated inflammation based on hemogram parameters, MCHC was found to be significantly lower in schizophrenia [27]. Furthermore, it has been found that low MCHC values predict depression in the long term and, in this regard, may be a better biomarker than hemoglobin [29]. It has been claimed that MCHC reflects tissue-level oxygenation better than total Hb concentration and that MCHC deficiency may also be associated with disruptions in antioxidant systems and increased pro-inflammatory cytokine levels [29]. In our study, similar to these findings, MCHC levels were lower despite similar hemoglobin levels indicating the potential significance of MCHC as a marker for TD. Further research is required to explore the implications and underlying mechanisms of these findings.

Red cell distribution width (RDW) is seen as an inflammatory marker that increases not only in many inflammatory and infectious diseases but also in psychiatric disorders such as schizophrenia [24]. In our study, although RDW was found to be high in the patient group, this difference was not statistically significant. This result does not support the hypothesis of inflammation in TD. The obtained result may be related to the limited number of patients. In future studies, retesting this hypothesis with larger sample sizes is recommended.

Parameters related to platelets and PCT were found to be significantly higher in the TD group. Platelets play a key role in modulating inflammatory processes by not only serving their primary role in homeostasis but also by secreting cytokines, chemokines, and other inflammatory mediators [30]. Additionally, platelets and neurons share similarities in the transport, metabolism, and release of various biogenic neurotransmitters, making platelets a diagnostic tool and an interesting research model in various psychiatric disorders [31]. Platelets exhibit many similarities with the neuronal monoamine system in the central nervous system. Abnormalities in platelets in depressive patients are mainly found in the serotonergic and noradrenergic systems [31]. However, platelets also have many dopamine receptors and can store and release dopamine [31]. The increase in platelet count observed in our study indicates increased platelet activation. This increase in activity may be related to an inflammatory response. In studies investigating inflammation in ASD, ADHD, and speech disorders, platelet values were also found to be significantly higher [14-16]. Considering that TD is classified under neurodevelopmental disorders like these [1], the elevation in platelet count might suggest a common etiological basis. TD is thought to involve complex interactions of multiple factors in its etiology. Dopamine neurotransmission, the hypothalamic-pituitary-adrenal (HPA) axis, associated inflammation, and changes in the cortico-striato-thalamo-cortical pathway are closely related [32,33]. Excessive dopamine in the striatum is believed to stimulate thalamocortical circuits [33]. The finding of high platelet counts in ADHD and OCD, both of which share similar neurobiological pathways with TD, supports this assumption [15,17,34]. Investigating the elevated platelet count in TD in future studies may serve as a guiding factor in shedding light on the underlying mechanisms of TD.

PCT is a measurement derived from the platelet count and the mean platelet volume. Physiologically, PCT is the most relevant parameter and is superior to the platelet count in estimating platelet status [15]. Previous studies have demonstrated a significant association between PCT and conditions such as autoimmune gastritis, acute coronary syndromes, inflammatory bowel diseases, and juvenile rheumatoid arthritis [35]. Elevated PCT has also been found in psychiatric disorders such as ADHD, depression, and bipolar disorder [15]. The elevated PCT found in our study may indicate a role for inflammation in TD.

Correlation analysis in our study revealed a negative correlation of disease duration with platelet and PCT. A similar negative correlation has been demonstrated in patients with hepatitis A infection [35]. However, explaining the mechanism behind this negative correlation within the scope of this study is not straightforward. Therefore, further research is needed to investigate the patterns of change in platelet count and PCT in TD cases.

In previous research, certain hematological parameters that are believed to be associated with inflammation, such as neutrophils, eosinophils, neutrophil to lymphocyte ratio (NLR), and platelet to lymphocyte ratio (PLR), were found to be higher in the TD group, although these differences did not reach statistical significance [17,21]. It is recommended that these parameters be retested for TD in future studies.

Our study has various strengths. The first one is the presence of a structured clinical interview, which is considered the gold standard for clinical diagnosis in studies. Additionally, the exclusion of patients with comorbid psychiatric disorders or those under psychopharmacological treatment, which could be confounding factors in terms of results, is among the other strengths of the study. 

Despite the strengths and significant results, our study has several limitations. The retrospective nature of the study is the most important limitation. Another limitation is the small sample size. Due to the limited number of cases, it was not possible to evaluate TD subtypes separately, and disease severity was not assessed. Other limitations include not comparing the results with other inflammatory mediators and not investigating iron, vitamin B12, and folate levels, which could potentially affect hematological parameters.

## Conclusions

Our study aimed to evaluate hemogram parameters in children and adolescents with TD to assess the potential role of inflammation in the disorder. To the best of our knowledge, this is the first study of its kind. The results of our study revealed several significant differences in hemogram parameters between TD patients and the control group.

These results may suggest the role of inflammation and/or other underlying mechanisms in TD. In light of these findings, it is advisable for future research to delve deeper into the mechanisms of inflammation in TD and its implications for diagnosis and treatment. In future studies, including more comprehensive inflammatory markers in larger and homogeneous groups may contribute to a deeper understanding of the relationship between inflammation and TD.
